# Entirely Extradural Meningioma in the Cervical Spine: A Case Report and Literature Overview

**DOI:** 10.7759/cureus.88062

**Published:** 2025-07-16

**Authors:** Ivo Kehayov, Georgi S Slavov, Georgi S Apostolov, Atanas Davarski, Borislav D Kitov

**Affiliations:** 1 Department of Neurosurgery, Medical University of Plovdiv, Plovdiv, BGR; 2 Department of Neurology, Medical University of Plovdiv, Plovdiv, BGR

**Keywords:** cervical surgery, extradural meningioma, postoperative outcome, spinal cord injury, spinal neurosurgery

## Abstract

Entirely extradural meningiomas of the cervical spine are exceptionally rare lesions arising from displaced arachnoid cells outside the dural sac. They typically present with neck pain, radicular symptoms, and myelopathic signs due to mass effect on the spinal cord. Imaging often reveals a well-defined, homogeneously enhancing epidural mass that may extend through the intervertebral foramina, mimicking more common lesions such as nerve sheath tumors or metastases. Here, we report a case of a crescent-shaped lesion encircling the right side of the cervical cord from C3 to C6, with foraminal extension at C4-C6. Surgical exploration via posterior laminectomy allowed for gross total resection, and histopathology confirmed a benign meningioma. The foraminal involvement highlighted the anatomical complexity of such lesions, which may at times necessitate subtotal resection to preserve neural structures. The patient’s postoperative course was notable for rapid symptom resolution and excellent functional recovery. Recognition of this entity is crucial for accurate diagnosis and optimal surgical management.

## Introduction

Spinal extradural meningiomas (SEMs) are rare and believed to arise from ectopic arachnoid cells displaced outside the dura mater during embryonic development, accounting for 0.8-1.8% of all meningiomas [1]. SEMs are usually round and confined entirely to the epidural space but can occasionally invade the intervertebral foramina or even grow into the paravertebral space. In these cases, they can dilate the intervertebral foramen and resemble schwannomas. Unlike the more common intradural meningiomas, which are typically slow-growing, well-circumscribed, and amenable to complete resection, SEMs may show more invasive behavior, pose greater challenges for total removal due to the involvement of the foramina and neurovascular structures, and may carry a higher risk of subtotal resection. Given the diagnostic challenges and surgical implications of extradural meningiomas with foraminal invasion, and considering that the vast majority of extradural spinal lesions are metastatic tumors, assuming the possibility of SEMs is essential for optimal preoperative planning and proper execution of surgical resection. Therefore, we report this case to highlight key imaging features and operative considerations.

Here, we present a rare case of an entirely extradural cervical meningioma surrounding the right side of the spinal cord as a cuff from the C3 to C6 level and invading the right C4, C5, and C6 intervertebral foramina.

## Case presentation

A 66-year-old woman with a history of atherosclerosis suffered from pain in the cervical region, which gradually irradiated to the right shoulder and the right arm, accompanied by numbness and tingling affecting the C5 and C6 dermatomes. She developed severe weakness of the right arm with difficulties in wrist extension and finger abduction. Conservative treatment administered at another medical institution did not result in significant improvement. Upon admission to our clinic, we established cervical vertebral syndrome with painful, limited movements in the neck. The neurological status showed mild signs of cervical myelopathy with impaired gait and severe weakness of the right arm (2/5), absent tendon reflexes, and difficulty extending the wrist and abducting the fingers. There was hyperesthesia and hyperalgesia across the right C5 and C6 dermatomes. Bowel and bladder control were preserved. Laboratory tests were within normal limits, except for slightly increased white blood cell count.

**Figure 1:**
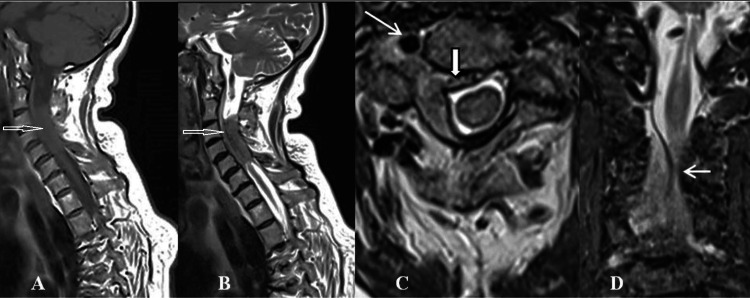
Preoperative MRI. A and B: Sagittal T1 and T2 images showing an extradural isointense tumor (thin hollow arrows). C: Axial projection C3 level. The tumor surrounds the medulla as a cuff (black and white thick arrow), penetrates the intervertebral foramen, and engulfs the right vertebral artery (white thin arrow). D: Coronal projection confirms the epidural localization of the lesion and the compression of the spinal cord (white thin arrow).

The MRI of the cervical spine detected an extradural isointense lesion on T2-weighted images extending from the C3 to C6 spinal level with dimensions of 39/19/6 mm. The lesion exhibited extensive growth involving the right C4, C5, and C6 neural foramina. The spinal cord was compressed from right to left and showed signs of ischemic myelopathy (Figure 1).

**Figure 2:**
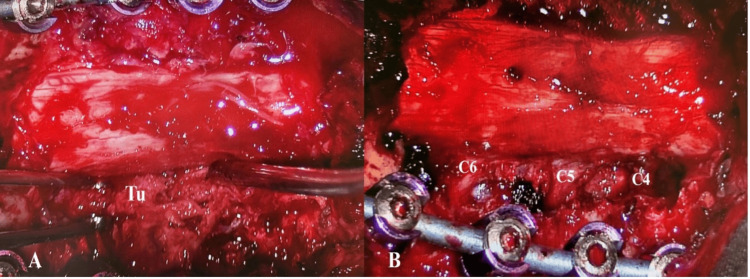
Intraoperative images. A: The extradural tumor (Tu) and the compression of the C4-C6 right nerve roots. B: Тhe decompression of the C4-C6 right nerve roots and the adjacent dural sac.

Based on the clinical picture and MRI findings, the patient was considered a surgical candidate. Given the planned multi-level laminectomies in accordance with the extension of the tumor from the C3 to C6 levels, we initially performed posterior trans-massa lateralis fixation in the C3-C6 segments to address postoperative spinal instability. After laminectomies from the C3 to C6 levels, a purely extradural tumor was encountered, which formed a right-sided cuff around the dural sac. The lesion involved the right C4, C5, and C6 neural foramina and had firm consistency. Using meticulous microsurgical technique, the tumor was removed to the maximum feasible extent, and the right C4, C5, and C6 nerve roots were adequately decompressed (Figure 2).

**Figure 3:**
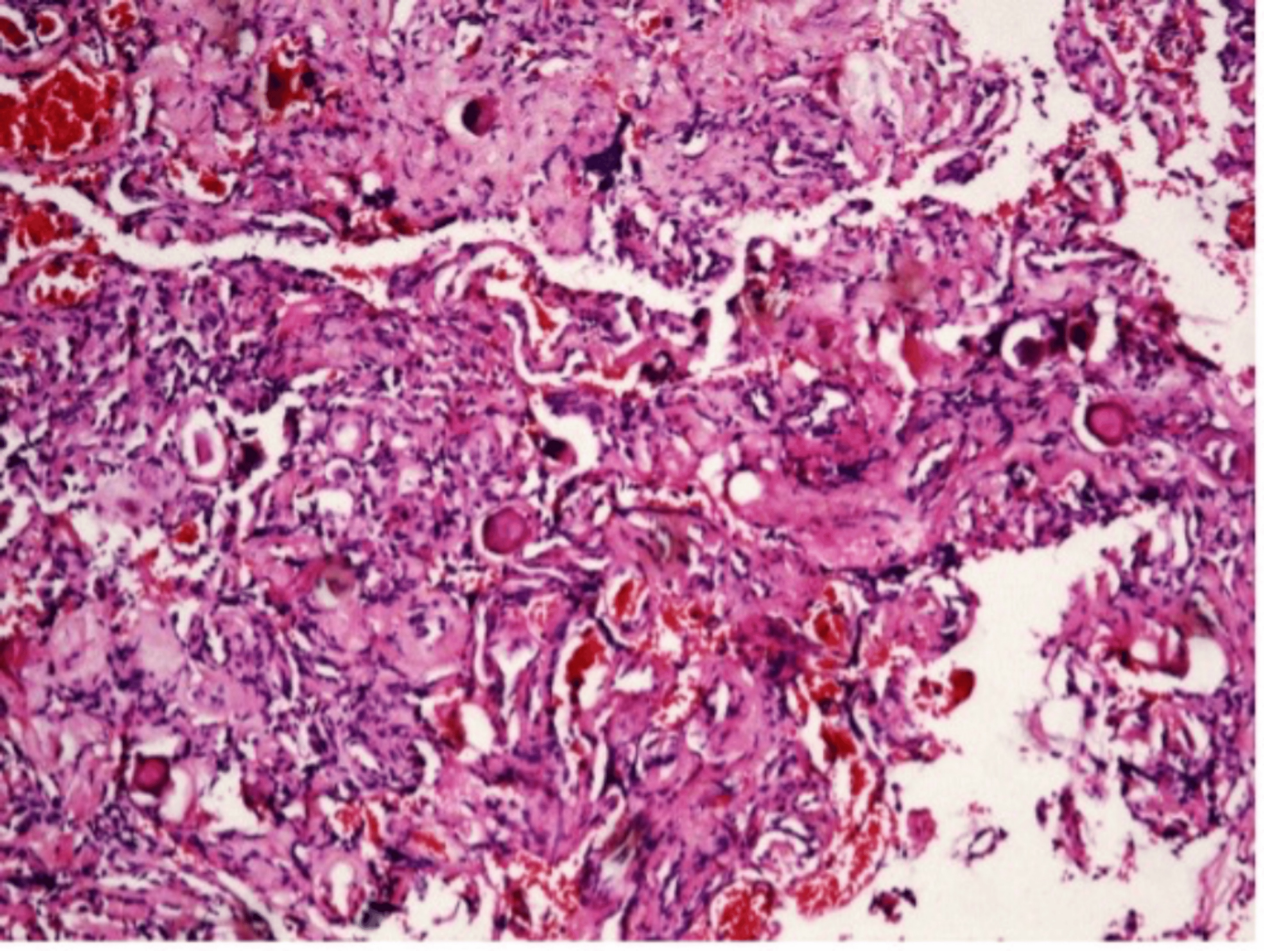
Histological representation. The tumor consists of a dense fibrous stroma with numerous psammoma bodies. Meningothelial cells are present but relatively sparse, arranged in clusters or single between the calcifications (hematoxylin and eosin, ×10).

**Figure 4:**
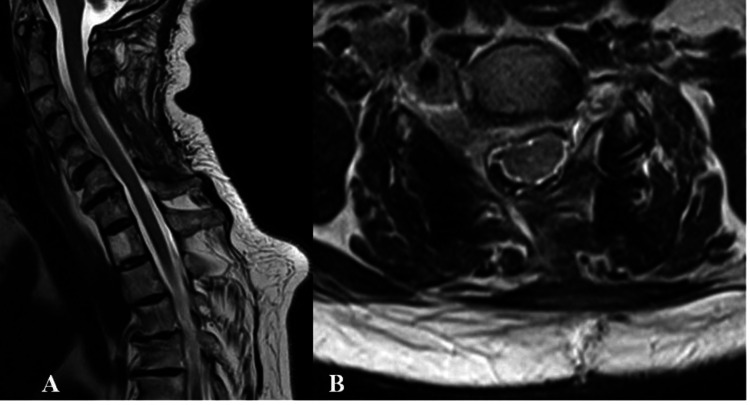
Follow-up cervical MRI after three months. Subtotal extirpation of the tumor lesion with adequate surgical decompression of the spinal cord. A: Sagittal projection with subtotal extirpation of the tumor lesion with adequate surgical decompression of the spinal cord. B: Axial projection with subtotal extirpation of the tumor lesion with adequate surgical decompression of the spinal cord.

The histological examination was consistent with the psammomatous variant of meningioma (Figure 3). The postoperative period was uneventful and without complications. A gradual reduction of the pain symptoms and myelopathic signs was documented. The neurological examination at the third postoperative month showed substantial gait improvement, reduction in the weakness of the right arm (4/5), and residual hypoesthesia along the C5 and C6 dermatomes. The follow-up MRI confirmed the subtotal tumor removal (Figure 4).

## Discussion

Extradural meningiomas account for approximately 2.5-3.5% of all spinal tumors [2]. According to Gezen et al., the most common location of extradural meningiomas is the thoracic spine (80%), followed by the cervical (16%) and lumbar (4%) regions [3]. Other sources report that the most common location of extradural meningiomas is the cervical region (61%-66%), followed by the thoracic region (44%-54%), and extremely rarely in the lumbar region (0.5%-2%) [1,4]. After a careful literature review, we found only 28 cases of purely extradural cervical meningiomas, some of which extend into the intervertebral foramina and beyond (Table 1).

**Table 1:** Cases of extradural meningiomas localized in the cervical region. *: Invasion of the intervertebral foramina.

Author (year)	Age (years)	Sex	Histology	Resection	Spinal level	Clinical presentation
Yokosako et al. (2025) [2]	51	Female	Psammomatous*	Subtotal	C2–C4	Myelopathy – limb paresis, spastic gait
Echalier et al. (2024) [5]	25	Male	Mixed*	Total	C5–T1 (brachial plexus)	Radiculopathy – upper limb sensory deficits and weakness
Nguyen et al. (2021) [6]	22	Female	Psammomatous	Total	C5–C8	Myelopathy – limb weakness and paresthesia
Lai et al. (2018) [7]	35	Male	Meningothelial*	Subtotal	Multi-segment cervical (C4–C7)	Neck pain, foraminal radiculopathy, vertebral artery encasement
Zhan et al. (2019) [8]	47	Female	Meningothelial	Subtotal	Single cervical level (likely C6)	Dumbbell mass, mild radicular pain
De Eulate‑Beramendi et al. (2019) [9]	42	Male	Meningothelial	Total (two-stage)	Cervical en-plaque	Sensorimotor deficits (upper limb/pain)
Sivaraju et al. (2017) [10]	50	Male	Psammomatous*	Subtotal	C5–C7	Spastic quadriparesis, brachial plexus involvement
Pant et al. (2017) [11]	50	Male	Meningothelial*	Subtotal	Cervical (C6–C7)	Neck pain and root compression
Yilmaz et al. (2016) [12]	17	Male	Unknown	Not operated	Cervical incidental	Asymptomatic
Bettaswamy et al. (2016) [13]	50, 41	Male/Male	Meningothelial*/Meningothelial	Subtotal/Total	Cervical (C3–C6)	Pain, numbness, mild weakness
Wu et al. (2014) [14]	42–50, varied	Mix	Mixed	Mixed	Cervical (various cases)	Mixed myeloradicular symptoms
Savardeker et al. (2014) [15]	35	Female	Meningothelial*	Subtotal	Cervical (C4–C6)	Radicular pain and paresis
Frank et al. (2008) [16]	45	Female	Psammomatous*	Subtotal	Cervical (C5–C6)	Neck/arm pain with radiculopathy
Yamada et al. (2007) [17]	22	Female	Meningothelial	Subtotal	Cervical (C6–C7)	Limb weakness and sensory changes
Takeuchi et al. (2005) [18]	50	Male	Meningothelial	Subtotal	Cervical (C4–C6)	Rapidly progressive myelopathy
Messori et al. (2002) [19]	14	Female	Meningothelial	Total	Cervical (C2–C5)	Neck pain, calcification-induced compression
Gamache et al. (2001) [20]	63	Female	Unknown	Subtotal	Cervical (C3–C5)	Sensory symptoms and mild weakness
Sato & Sze (1997) [21]	39	Male	Atypical	Subtotal	Cervical (C5–C6)	Pain, mild myelopathy
Chen et al. (1992) [22]	14	Female	Meningothelial	Total	Cervical (C1–C3)	Neck pain and root symptoms
Our case (2025)	66	Female	Psammomatous*	Subtotal	C3–C6	Radiculomyelopathy – cord compression + foraminal radicular pain

Unlike intradural extramedullary meningiomas, which are most commonly seen between the fifth and seventh decades, and the female-to-male ratio ranges from 4:1 to 14:1, the mean age of patients with cervical extradural meningiomas is 38.48 years (14-66 years), with a slight female predominance of 1.2:1 [23].

Most likely, extradural meningiomas arise from ectopic or detached arachnoid tissue around the periradicular sheath of the nerve roots, where the spinal leptomeninges fuse directly into the dura [24]. There is a hypothesis that the periradicular dura mater, which is thinner, may contain traces of embryonic arachnoid mater and villi in its superficial layer. This may lead to the development of epidural meningiomas in the vicinity of the nerve root [24]. Another hypothesis suggests that extradural meningiomas arise from islands of arachnoid tissue that have migrated into the extradural space [25].

Extradural meningiomas are most often benign, often with a long clinical history until diagnosis, with the average duration of symptoms before diagnosis ranging from three weeks to ten years, and in our case, six weeks [1]. The most common symptoms include motor deficits (57%) and sensory deficits (50%), and in rare cases of sphincter disorders, which is also confirmed by the clinical presentation of our patient [13]. They have more aggressive growth and a greater likelihood of clinical deterioration [9].

Although most cervical extradural meningiomas are confined to the epidural space, in some cases, they invade the intervertebral foramina and paravertebral structures, as in our case [2]. Histological types of extradural meningiomas include meningothelial, fibroblastic, transitional, and psammomatous, with meningothelial (44.8%) and psammomatous (31.2%) being the most common [1].

MRI before and after contrast administration is the gold standard for the diagnosis of SEM, which allows both to establish the localization of the tumor and its relationship with the surrounding bone and nerve structures, as well as to develop optimal preoperative planning. Typically, the MRI images of SEM are isointense in the T1 sequence and minimally increased intensity in the T2 sequence, and upon injection of contrast material, the latter accumulates relatively homogeneously within the tumor mass [24]. In cases of extradural lesions, especially when there is invasion through the intervertebral foramen, a metastatic process or extradural lymphoma may be suspected. The differential diagnosis also includes nerve sheath tumors (schwannoma or neurofibroma), chordoma, synovial cyst, or infectious process [26]. MRI of spinal schwannomas and neurofibromas are similar, being hypointense in T1 and moderately hyperintense in T2 [27]. In these cases, focal areas of lower intensity associated with intralesional hemorrhage or collagen deposition, as well as regions of higher intensity corresponding to cystic regions, may be observed in T2 sequences [27]. The injected contrast agent usually accumulates inhomogeneously in the tumor or its periphery [27]. There is a diagnostic test for schwannomas that is based on several parameters: craniocaudal location, presence of hyperintense and/or heterogeneous signal on T2-weighted MRI, and heterogeneous accumulation of contrast agent in the tumor [28]. According to De Verdelhan et al., this test supports the diagnosis of schwannoma with a sensitivity of 96.4% and a specificity of 83.3% [28].

In T1-weighted MRI, features of chordoma are isointense to the bone marrow, while SEM features are isointense to the spinal cord and do not involve the vertebral body [26]. Synovial cysts are adjacent to the joint, spherical, and usually no larger than 1 to 2 cm [26].

The treatment of choice for extradural meningiomas is surgical resection, but total removal of the lesion can be challenging, especially in cases where the tumor surrounds the spinal cord in en-plaque fashion and/or extends through the intervertebral foramina to reach and engulf the vertebral artery, as in our case [29]. The literature review shows that total extirpation can be achieved in only 27.6% of cases (Table 1). Subtotal resection is associated with a higher recurrence rate compared with intradural meningiomas [17].

Given the challenges of achieving gross total resection in cases with foraminal extension or vertebral artery encasement, as in our case, adjuvant radiotherapy may be considered to reduce the risk of recurrence. Although most extradural meningiomas are WHO grade I, subtotal resection is associated with higher recurrence rates, and radiotherapy can offer effective local control when reoperation is not feasible.

## Conclusions

Extradural meningiomas are extremely rare, and differential diagnosis is challenging, especially in cases with invasion of the intervertebral foramen and paravertebral muscles. In these cases, total extirpation of the lesion is rarely achieved, despite the use of advanced surgical techniques and neuromonitoring. Although optimal decompression of the neural structures is achieved by subtotal resection, long-term clinical and imaging follow-up of the patients is imperative, especially considering their relatively young age.

## References

[1] Balasubramanian K, Zuccato JA, Kharbat AF, Janssen C, Gonzalez NM, Dunn IF (2024). Primary extradural meningioma: a systematic review of diagnostic features, clinical management, and surgical outcomes. Cancers (Basel).

[2] Yokosako S, Hirasawa M, Kubota Y (2025). A case of cervical epidural meningioma with atypical image findings. Surg Neurol Int.

[3] Gezen F, Kahraman S, Canakci Z, Bedük A (2000). Review of 36 cases of spinal cord meningioma. Spine (Phila Pa 1976).

[4] Shui C, Turchini J, Davies M (2021). Purely extradural spinal meningioma: a case report and literature review. Surg Neurol Int.

[5] Echalier C, Chevrier B, Gros P, Teboul F, Goubier JN (2024). Case report of a primary ectopic extradural and extraspinal meningioma of the brachial plexus. Neurochirurgie.

[6] Nguyen BQ, Tran DD, Dang TC, Mai TD, Pham HD, Truong VT (2021). Cervical intra-extradural meningioma with en-plaque, dumbbell-shaped, and an unusual calcified pattern in a young patient. Surg Neurol Int.

[7] Lai AL, Salkade PR, Chuah KL, Sitoh YY (2018). Extradural cervical spinal meningioma mimicking malignancy. J Radiol Case Rep.

[8] Zhan Z, Yan X, Nie W, Ding Y, Xu W, Huang H (2019). Neurofibroma and meningioma within a single dumbbell-shaped tumor at the same cervical level without neurofibromatosis: a case report and literature review. World Neurosurg.

[9] de Eulate-Beramendi SA, Piña-Batista KM, Rial-Basalo JC (2019). Extradural en-plaque spinal lipomatous meningioma: a case report and literature review. Surg Neurol Int.

[10] Sivaraju L, Thakar S, Ghosal N, Hegde AS (2017). Cervical en-plaque extradural meningioma involving brachial plexus. World Neurosurg.

[11] Pant I, Gautam VK, Kumari R, Chaturvedi S (2017). Spinal tumour: primary cervical extradural meningioma at an unusual location. J Spine Surg.

[12] Yilmaz A, Kizilay Z, Sair A, Avcil M, Ozkul A (2016). Spontaneous regression of an incidental spinal meningioma. Open Access Maced J Med Sci.

[13] Bettaswamy G, Ambesh P, Das KK (2016). Extradural spinal meningioma: revisiting a rare entity. J Craniovertebr Junction Spine.

[14] Wu L, Yang T, Deng X (2014). Spinal extradural en plaque meningiomas: clinical features and long-term outcomes of 12 cases. J Neurosurg Spine.

[15] Savardekar A, Chatterjee D, Chatterjee D, Dhandapani S, Mohindra S, Salunke P (2014). Totally extradural spinal en plaque meningiomas - diagnostic dilemmas and treatment strategies. Surg Neurol Int.

[16] Frank BL, Harrop JS, Hanna A, Ratliff J (2008). Cervical extradural meningioma: case report and literature review. J Spinal Cord Med.

[17] Yamada S, Kawai S, Yonezawa T, Masui K, Nishi N, Fujiwara K (2007). Cervical extradural en-plaque meningioma. Neurol Med Chir (Tokyo).

[18] Takeuchi H, Kubota T, Sato K, Hirose S (2006). Cervical extradural meningioma with rapidly progressive myelopathy. J Clin Neurosci.

[19] Messori A, Rychlicki F, Salvolini U (2002). Spinal epidural en-plaque meningioma with an unusual pattern of calcification in a 14-year-old girl: case report and review of the literature. Neuroradiology.

[20] Gamache FW Jr, Wang JC, Deck M, Heise C (2001). Unusual appearance of an en plaque meningioma of the cervical spinal canal. A case report and literature review. Spine (Phila Pa 1976).

[21] Sato N, Sze G (1997). Extradural spinal meningioma: MRI. Neuroradiology.

[22] Chen HJ, Lui CC, Chen L (1992). Spinal epidural meningioma in a child. Childs Nerv Syst.

[23] Kitov B, Apostolov G, Davarski A, Kehayov I, Kilova K (2022). Analysis of characteristics and surgical outcome of intradural extramedullary tumors - a retrospective cohort study of 52 patients. Folia Med (Plovdiv).

[24] Redhu R, Pavithra HN (2024). Spinal extradural meningioma: report of two cases. J Craniovertebr Junction Spine.

[25] Santiago BM, Rodeia P, Cunha E Sa M (2009). Extradural thoracic spinal meningioma. Neurol India.

[26] Dang DD, Mugge LA, Awan OK, Gong AD, Fanous AA (2024). Spinal meningiomas: a comprehensive review and update on advancements in molecular characterization, diagnostics, surgical approach and technology, and alternative therapies. Cancers (Basel).

[27] Apostolov G, Kitov B, Poryazova E, Kehayov I (2021). Sporadic spinal schwannomas and neurofibromas - a review. Folia Med (Plovdiv).

[28] De Verdelhan O, Haegelen C, Carsin-Nicol B (2005). MR imaging features of spinal schwannomas and meningiomas. J Neuroradiol.

[29] Zhang LH, Yuan HS (2018). Imaging appearances and pathologic characteristics of spinal epidural meningioma. AJNR Am J Neuroradiol.

